# Is arginase a potential drug target in tobacco-induced pulmonary endothelial dysfunction?

**DOI:** 10.1186/s12931-015-0196-4

**Published:** 2015-03-28

**Authors:** Priscilla Henno, Christelle Maurey, Françoise Le Pimpec-Barthes, Philippe Devillier, Christophe Delclaux, Dominique Israël-Biet

**Affiliations:** Sorbonne Universités, UPMC Université Paris 06, Paris, France; Département Physiologie-Algologie-Somnologie, Unité Fonctionnelle de Somnologie et Fonction Respiratoire, AP-HP, Hôpital Saint Antoine, 75012 Paris, France; Laboratoire de Pharmacologie Respiratoire UPRES EA 220, Hôpital Foch, 92150 Suresnes, France; Ecole Nationale Vétérinaire d’Alfort, Unité de Médecine, Université Paris-Est, 94700 Maisons-Alfort, France; Sorbonne Paris Cité, Université Paris-Descartes, Paris, France; Service de Chirurgie Thoracique, AP-HP, Hôpital Européen Georges Pompidou, 75015 Paris, France; Université Versailles Saint-Quentin en Yvelines, UFR Sciences de la Santé Simone Veil, Montigny le Bretonneux, France; Service de Physiologie, Explorations Fonctionnelles Respiratoires et du Sommeil, AP-HP, Hôpital Européen Georges Pompidou, 75015 Paris, France; Service de Pneumologie, AP-HP, Hôpital Européen Georges Pompidou, 75015 Paris, France

**Keywords:** Endothelial dysfunction, Arginase, Tobacco smoking

## Abstract

**Background:**

Tobacco-induced pulmonary vascular disease is partly driven by endothelial dysfunction. The bioavailability of the potent vasodilator nitric oxide (NO) depends on competition between NO synthase-3 (NOS3) and arginases for their common substrate (L-arginine). We tested the hypothesis whereby tobacco smoking impairs pulmonary endothelial function via upregulation of the arginase pathway.

**Methods:**

Endothelium-dependent vasodilation in response to acetylcholine (Ach) was compared *ex vivo* for pulmonary vascular rings from 29 smokers and 10 never-smokers. The results were expressed as a percentage of the contraction with phenylephrine. We tested the effects of L-arginine supplementation, arginase inhibition (by N(omega)-hydroxy-nor-l-arginine, NorNOHA) and NOS3 induction (by genistein) on vasodilation. Protein levels of NOS3 and arginases I and II in the pulmonary arteries were quantified by Western blotting.

**Results:**

Overall, vasodilation was impaired in smokers (relative to controls; p < 0.01). Eleven of the 29 smokers (the ED^+^ subgroup) displayed endothelial dysfunction (defined as the absence of a relaxant response to Ach), whereas 18 (the ED^−^ subgroup) had normal vasodilation. The mean responses to 10^−4^ M Ach were −23 ± 10% and 31 ± 4% in the ED^+^ and ED^−^ subgroups, respectively (p < 0.01). Supplementation with L- arginine improved endothelial function in the ED^+^ subgroup (−4 ± 10% vs. -32 ± 10% in the presence and absence of L- arginine, respectively; p = 0.006), as did arginase inhibition (18 ± 9% vs. -1 ± 9%, respectively; p = 0.0002). Arginase I protein was overexpressed in ED^+^ samples, whereas ED^+^ and ED^−^ samples did not differ significantly in terms of NOS3 expression. Treatment with genistein did not significantly improve endothelial function in ED^+^ samples.

**Conclusion:**

Overexpression and elevated activity of arginase I are involved in tobacco-induced pulmonary endothelial dysfunction.

## Background

Tobacco smoking can induce pulmonary vascular remodelling – even in individuals with normal or slightly impaired lung function [1]. This remodelling can lead to increased pulmonary vascular resistance and then pulmonary hypertension (PH). As a well characterized feature of end-stage chronic obstructive pulmonary disease (COPD) [2], PH can also develop in milder forms of COPD and is acknowledged to be a significant clinical issue with a strong, negative impact on the prognosis [3]. Pulmonary endothelial dysfunction is thought to be an early pathophysiological determinant of vascular remodelling. The condition is present in end-stage COPD [4] but has also been observed in milder forms of COPD and in smokers without COPD [5]. The mechanisms of pulmonary endothelial dysfunction have not been well elucidated but may be based (at least in part) on (i) imbalance between the production of vasoconstrictive and vasodilatory factors and (ii) low bioavailability of nitric oxide (NO) [6], which is a potent vasodilator and decreases vascular smooth muscle cell proliferation. The production of NO by endothelial nitric oxide synthase (NOS3) is dependent on the balance between the expression and/or activity of arginases and NOS3. In the vasculature, NOSs and arginases compete for their common substrate L-arginine (L-arg). Elevated arginase activity reduces the availability of L-arg for NOS3 and thus decreases the production of NO. In addition, arginases I and II (which are also expressed by endothelial cells) produce urea and L-ornithine [7,8]. L-ornithine metabolism produces polyamines and proline, which are involved in pulmonary vascular remodelling [9]. Consequently, arginases and NOS may have opposing effects on vascular tone and tissue remodelling [10]. Over the last few years, it has become increasingly clear that arginases have a harmful role in systemic vascular conditions in animal models and in humans (including atherosclerosis, coronary artery disease, myocardial ischemia-reperfusion, diabetes mellitus, heart failure, hypertension and ageing) [11-18]. Cigarette smoke can increase arginase activity and protein expression in systemic vessels or in the lung [19-21]. Although arginases are also expressed in the pulmonary vasculature, [22,23] there are no literature data on the latter’s role in tobacco-induced pulmonary endothelial dysfunction in humans.

Hence, we decided to test the hypothesis whereby tobacco smoking impairs pulmonary endothelial function through upregulation of the arginase pathway.

## Methods

We obtained explants from current smokers, ex-smokers or never-smokers undergoing lung resection for lung cancer in a major university hospital (Hôpital Européen George Pompidou, Paris, France). The study’s objectives and procedures were approved by the local independent ethics committee, and all patients gave their written, informed consent to participation in the study.

### Tissue preparation

Immediately after excision, lung tissue samples were placed in Krebs-Henseleit solution (mM: 120 NaCl, 4.7 KCl, 2.5 CaCl_2_, 1.2 MgCl_2_, 15 NaHCO_3_, 1.2 KH_2_PO_4_, 11 D-glucose and 10 HEPES, pH 7.4) and immediately transported to our laboratory. After intralobar arteries had been carefully dissected free of parenchyma and adhering connective tissue, several rings (3 to 5 mm in length, and 1.5 to 2 mm in internal diameter) were prepared from a single artery. Some of the rings were used immediately for pharmacological studies, whereas others were snap-frozen and stored in liquid nitrogen for subsequent protein extraction.

Endothelial function was evaluated by the cumulative acetylcholine (Ach) dose response curve for pulmonary artery rings isolated from smokers or never-smokers. Under our experimental conditions, endothelial dysfunction was defined as an impaired response to Ach (such as a lack of relaxation, or even contraction).

### The pharmacological experiments

Arterial rings were mounted in bath organs, as previously described [5]. Briefly, rings were suspended on tissue hooks in 5 ml organ baths containing Krebs-Henseleit solution at 37°C and bubbled with 95% O_2_ and 5% CO_2_. Each preparation was connected to a force displacement transducer (Statham UF-1) and changes in isometric tension were recorded. An initial tension of 1 g was applied to the rings, which were then left to equilibrate for 30 minutes (with regular changes in fresh Krebs-Henseleit solution) until a stable resting tension (RT1) was obtained. The rings’ responsiveness was confirmed by adding KCl (to 40 mM), which induced contraction. Viable rings were then washed until full relaxation had occurred (resting tension 2, RT2), and were left to rest for 20 minutes. The rings were then precontracted with L-phenylephrine (PE) dichloride (10^−5^ M) to obtain a stable plateau of contraction. Serial dilutions of Ach chloride were then added, in order to establish a cumulative dose response curve (10^−10^ to 10^−4^ M). Relaxation in response to Ach was expressed as a percentage of the contraction induced by PE. A contractile response to Ach was expressed as a negative value. Endothelium-independent relaxation was assessed by measuring the response to sodium nitroprusside 10^−5^ M at the end of each experiment.

For each patient, some rings were pre-treated with various drugs for 30 minutes after PE precontraction. To test the hypothesis whereby NOS lacked substrate, we supplemented the rings with L-arg (10^−3^ M). To evaluate the role of arginases in pulmonary vasoactivity, we assessed the effects of the arginase antagonist N(omega)-hydroxy-nor-l-arginine (NorNOHA; 10^−5^ M). Lastly, we tested the effect of the NO potentiator genistein (10^−6^ M). The concentrations of these drugs producing 50% of the maximum effect were determined in preliminary experiments (data not shown).

All drugs were purchased from Sigma (St. Louis, MO), with the exception of Ach (provided by Pharmacie Centrale des Hôpitaux, Paris, France).

All experiments were performed in duplicate. The inter-ring variability was always below 10%.

### Western blot analyses

Nitric oxide synthase 3 and arginases 1 and 2 were assayed in homogenized extracts of pulmonary arteries, as previously described [5]. Total proteins were extracted with a lysis buffer (10 mM Tris–HCl pH 7.4, 50 mM NaCl, 0.1% NP-40, and 20% antiprotease cocktail) and were measured with a bicinchoninic acid protein assay kit (Pierce) on a microplate, according to the manufacturer’s instructions. Total proteins (30 μg/lane) were separated by electrophoresis on a 10% sodium dodecyl sulphate–polyacrylamide gel, transferred onto nitrocellulose membranes and immunodetected. Nonspecific binding was blocked by incubation with 10% milk powder in Tris-buffered saline for 1 h at room temperature. The blots were incubated with a rabbit polyclonal antibody against NOS3 (sc-654, 1:150 dilution, Santa Cruz Biotechnology, Inc., Dallas, TX) or goat polyclonal antibodies against arginases I or II (sc-18351 and sc 18360, 1:200 dilution, Santa Cruz Biotechnology) followed by biotinylated secondary antibodies: anti-rabbit IgG peroxidase conjugate (sc-2004, 1:1,000 dilution; Santa Cruz Biotechnology) or anti-goat IgG peroxidase conjugate (sc-2922, 1:1,000 dilution; Santa Cruz Biotechnology). Membranes were subsequently stripped and reprobed with a mouse monoclonal antibody against β-actin (AANO2; dilution: 1:1,000; Cytoskeleton, Inc., Denver, CO). The proteins were detected with an enhanced chemiluminescence (ECL) kit and high-performance chemiluminescence film (GE Healthcare, Aulnay sous Bois, France). The intensities of protein staining on the immunoreactive Western blot bands were analyzed with ImageJ image analysis software. The relative amounts of immunoreactive proteins were calculated by dividing the scanning unit value by the respective value of β-actin protein (probed with the primary anti-β-actin antibody) and expressed in arbitrary units.

### Statistical analysis

Results are expressed as the mean ± standard error of the mean (SEM), except where otherwise specified. Data were analysed with NCSS9 (NCSS, LLC. Kaysville, UT) and GraphPad Prism version 5.00 for Windows (GraphPad Software, San Diego, CA) For intergroup comparisons, a non-parametric analysis of variance (ANOVA) was followed by Dunn’s test for multiple comparisons. For comparisons of condition (i.e. Ach dose–response curves in the presence and absence of another drug), a repeated measures ANOVA was followed by a Tukey-Kramer test for multiple comparisons. Fischer’s exact test or Mann–Whitney test was used for categorical variables. A p value <0.05 was considered to be statistically significant.

## Results

### Subjects

Explants were obtained from 29 current smokers/ex-smokers and 10 never-smokers. Smoking history was the only demographic or clinical factor that differed significantly when comparing smokers and never-smokers (Table 1).

**Table 1:** **Clinical characteristics of the study population**

**Characteristics**	**Smokers**	**Never-smokers**	**p**
	**(n = 29)**	**(n = 10)**	
Age, years (median, range)	63 (44–78)	55 (49–73)	0.11
Male:female ratio	23:6	7:3	0.16
Tobacco, pack-years (mean, range)	48 (10–120)	NA	
Current smokers (n=)	15	NA	
FEV1, % predicted (mean, range)	88 (66–118)	92 (82–103)	0.33
OLD (n=)	2	0	1

NA: not appliable; FEV1: forced expiratory volume in 1 second; OLD: presence of an obstructive pulmonary disease, defined as post-bronchodilator FEV1/forced vital capacity < 70%.

### Tobacco smoking impairs the relaxant response of the pulmonary artery

**Figure 1:**
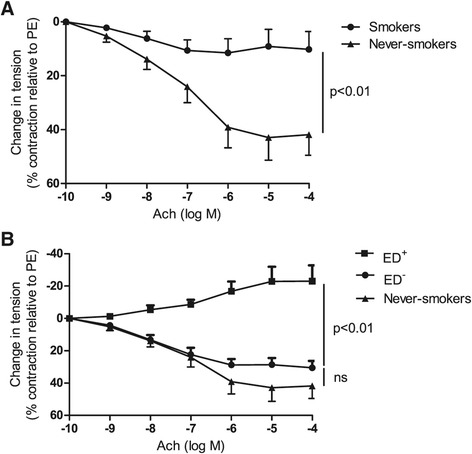
**Pulmonary endothelial function, represented as cumulative Ach dose response curves in pulmonary artery rings from smokers (n = 29) and never-smokers (n = 10).** Smokers had impaired relaxation in response to Ach, when compared with never-smokers (p < 0.01; panel **A)**. The smokers were divided into two subgroups according to the presence (ED^+^, n = 11) or absence (ED^−^, n = 18) of pulmonary endothelial dysfunction (p < 0.01; panel **B)**.

The vasodilatory response to Ach was strongly altered in smokers, when compared with never-smokers (10 ± 7% vs. 42 ± 8% at 10^−4^ M Ach, respectively, p < 0.01, Figure 1, panel A). Closer analysis of responses of the rings from the smokers enabled us to distinguish between two subgroups: 11 patients exhibited endothelial dysfunction as defined above (the ED^+^ subgroup), whereas the remaining 18 exhibited a relaxant response (forming the ED^−^ subgroup) similar to that seen in never-smokers (−23 ± 10% vs. 31 ± 4% and 42 ± 8% at 10^−4^ M Ach in the ED^+^ subgroup, the ED^−^ subgroup and never-smokers, respectively; p < 0.01, Figure 1, panel B). The ED^+^ vs. ED^−^ subgroups did not differ significantly in terms of baseline tensions or peak vasoconstriction in response to PE (RT1: 0.9 ± 0.1 g vs. 0.9 ± 0.1 g (ns), respectively; RT2: 0.8 ± 0.1 g vs. 0.9 ± 0.1 g (ns), respectively; PE-induced tension: 1.2 ± 0.2 g vs. 1.4 ± 0.1 g, respectively; data not shown). SNP 10^−5^ M induced a relaxant response in all patients, including those with an endothelial dysfunction, discarding the fact that the lack of endothelium-dependant response could be due to the damage of the pulmonary artery during its manipulation (data not shown). In neither subgroup was the response to Ach correlated with age, gender, cumulative tobacco smoking, COPD, forced expiratory flow in 1 second (FEV1), systolic pulmonary artery pressure (as measured by echocardiography, when available) or other possible confounders of endothelial dysfunction, such as hypertension, hypercholesterolemia, ongoing treatment with statins or vasodilators, diabetes mellitus, and prior treatment with cytotoxic drugs (Table 2).

**Table 2:** **Clinical characteristics of patients according to the presence or absence of endothelial dysfunction**

**Characteristics**	**Smokers**		**p**
	**ED** ^**+**^ **(n = 11)**	**ED** ^**−**^ **(n = 18)**	
Age, years (median, range)	63 (44–77)	62 (48–78)	0.86
Male:Female ratio	7:4	16:2	0.11
Tobacco, pack-years (mean, range)	54 (30–120)	46 (10–90)	0.74
Current smokers (n=)	5	10	0.96
FEV1, % predicted (mean, range)	79 (67–101)	92 (66–118)	0.06
OLD (n=)	0	2	0.51
GOLD 1 (n=)	0	1	1
GOLD 2 (n=)	0	1	1
Prior chemotherapy (n=)	2	4	1
Hypercholesterolemia (n=)	2	1	0.53
Hypertension (n=)	2	5	0.68
Diabetes mellitus (n=)	1	3	1
Treatment by statin (n=)	1	5	0.36
Vasodilating treatment (n=)	1	3	1

ED^+^: endothelial dysfunction; ED^−^: no endothelial dysfunction; FEV1: forced expiratory volume in 1 second; OLD: presence of an obstructive pulmonary disease, defined as post-bronchodilator FEV1/forced vital capacity < 70%.

All of the 11 ED^+^ patients had undergone pre-operative echocardiography and none had displayed an elevated systolic pulmonary artery pressure at rest.

### L-arginine supplementation improves endothelium-dependent function

**Figure 2:**
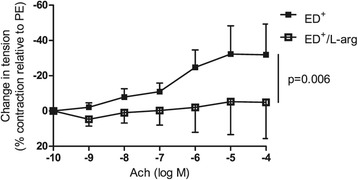
**Effect of L-arg (10** ^**−3**^ **M) supplementation on endothelial function.** L-arg supplementation improved endothelial function in ED^+^ samples (n = 6; p = 0.006).

To investigate whether endothelial dysfunction could be explained by a lack of the NOS substrate L-arg, we measured the ED^+^ samples’ Ach dose–response curves in the presence and absence of substrate supplementation (n = 6). Supplementation with L-arg (10^−3^ M) significantly improved endothelial function but did not restore a normal vasodilatory response (−4 ± 10% vs. -32 ± 10% at 10^−4^ M Ach in the presence and absence of L-arg, respectively; p = 0.006; Figure 2).

### Arginase inhibition restores endothelium-dependent dilation

**Figure 3:**
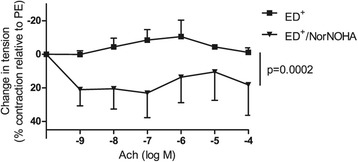
**Effect of arginase inhibition by NorNOHA (10** ^**−5**^ **M) on endothelial function.** NorNOHA improved endothelial function in ED^+^ samples (n = 4) by restoring a vasodilatory response to Ach (p = 0.0002).

We tested the effect of the arginase inhibitor NorNOHA (10^−5^ M) on the ED^+^ samples’ response to Ach (n = 4). Arginase inhibition significantly improved endothelial function and restored the relaxant response to Ach (18 ± 9% vs. -1 ± 9% at 10^−4^ M Ach in the presence and absence of NorNOHA, respectively; p = 0.0002; Figure 3).

#### Effect of genistein on endothelial dysfunction

**Figure 4:**
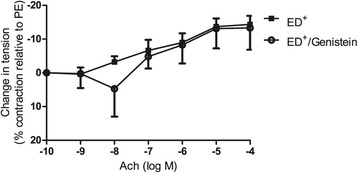
**Effect of genistein (10** ^**−6**^ **M) on endothelial function.** Genistein did not improve endothelial function in ED^+^ patients (n = 4).

The phytoestrogen genistein can potentially enhance NO production by activating NOS3. We sought to determine whether the compound could improve the vasodilatory response in ED^+^ subjects (n = 4). Genistein (10^−6^ M) did not improve this response (−13 ± 7% vs. -14 ± 3% at 10^−4^ M Ach in the presence and absence of genistein, respectively, NS) (Figure 4).

### NOS and arginase expression

**Figure 5:**
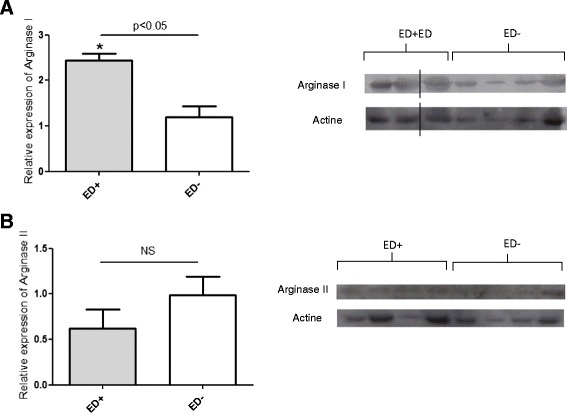
**Protein expression of arginases I and II in pulmonary artery samples from ED** ^**+**^ **and ED** ^**−**^ **patients.** Arginase I protein expression (as analysed by Western blotting) was higher in ED^+^ patients (n = 3) than in ED- patients (n = 4), p<0.05; panel **A**. Arginase II protein expression was similar in ED^+^ (n = 4) and ED^−^ patients (n = 4); panel **B**. The vertical line indicates that the gel was cut.

**Figure 6:**
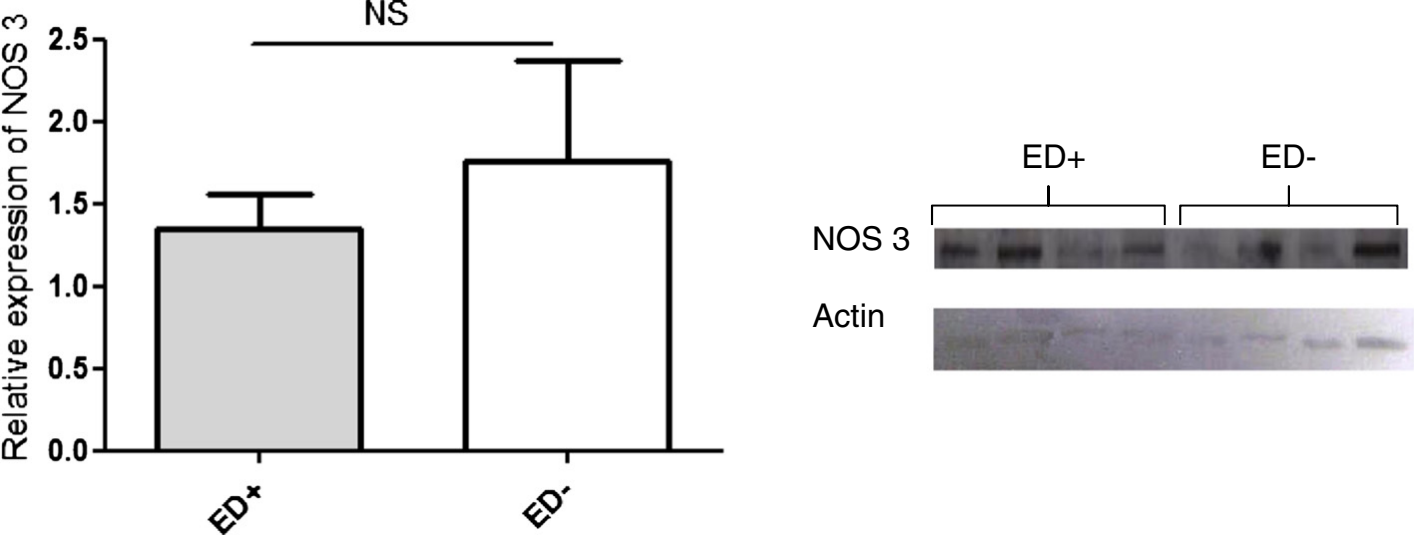
**Expression of NOS3 in pulmonary artery samples from ED** ^**+**^ **and ED** ^**−**^ **patients.** NOS3 protein expression was similar in ED^+^ (n = 4) and ED^−^ (n = 4) patients.

In pulmonary artery samples analysed with Western blotting, arginase I protein expression was significantly higher in ED^+^ patients (n = 3) than in ED^−^ patients (n = 4) (mean relative expression: 2.45 ± 0.15 vs. 1.19 ± 0.25, respectively; p < 0.05; Figure 5, panel A). In contrast, levels of arginase II expression were similar the ED^+^ (n = 4) and ED^−^ subgroups (n = 4) (Figure 5, panel B). Likewise, the ED^+^ and ED^−^ subgroups did not differ significantly in terms of the expression of NOS3 in pulmonary artery rings (mean relative expression: 1.34 ± 0.2 vs. 1.76 ± 1.2, respectively, NS) (Figure 6).

## Discussion

We found that the impairment in pulmonary vasodilation observed in a significant proportion of smokers may be explained by increased arginase I protein expression in the pulmonary artery. L-arg supplementation reduced the endothelial dysfunction, and arginase inhibition restored a normal vasodilatory, endothelium-dependent response in ED^+^ patients. Lastly, we confirmed our previous report of the high prevalence of pulmonary endothelial dysfunction in smokers (38% in the present study) [5], even in those with normal lung function. Indeed, our smokers and nonsmokers were quite similar in terms of clinical characteristics (and cardiovascular risk factors in particular), and there were only two smokers in the study population with overt, moderate COPD, Global Initiative for Obstructive Lung Disease (GOLD) stage 2. The other smokers had normal lung function. Taken as a whole, our present data strengthen the general hypothesis whereby tobacco smoking has a direct, harmful influence on the pulmonary vasculature.

In terms of putative mechanisms, our study focused on the NO pathway because changes in NO bioavailability are thought to have a major role in endothelial dysfunction [6]. Nitric oxide acts as an endothelial central signalling molecule [24] that controls vascular tone and structure by decreasing vascular smooth muscle cell proliferation [25,26]. A decreased NO bioavailability, as we hypothesize in smokers exhibiting an abnormal vasorelaxation (ED^+^ subjects) might partly result from a limitation in substrate [6,21,15]. Indeed, arginase and NOS compete for their common substrate, L-arginine, and an overactive arginase might limit substrate availability for NOS and therefore NO production. In addition, an increased oxidative stress might also partly account for the vascular dysfunction showed in this study. Our experiments revealed that NO bioavailability was impaired in ED^+^ subjects as a result of absolute and relative substrate deficiencies. Indeed, the arginase inhibitor NorNOHA was able to partly restore a relaxant response, suggesting that ED^+^ subjects have elevated arginase activity. This can be explained by the elevated vascular arginase I protein expression observed in ED^+^ samples. Taken as a whole, these results suggest that NO production by NOS3 is limited by competition with arginase I for the common substrate L-arg, which ultimately leads to a relative substrate deficiency. Enhanced arginase activity has been associated with the systemic endothelial dysfunction observed in various conditions. Some cell-based and preclinical studies using arginase inhibitors have provided convincing evidence for arginase’s harmful effects in cardiovascular diseases [15,18] in which arginase expression is upregulated [17,27]. Although data for the pulmonary circulation are scarce, harmful effects of arginase upregulation and/or of low L-arg levels have also been demonstrated in models of primary or secondary PH [22,28-32]. To the best of our knowledge, our study is the first to have demonstrated the role of increased arginase activity in tobacco associated-pulmonary endothelial dysfunction. Increased arginase activity has also been associated with airway hyperresponsiveness [33,34]; we previously demonstrated that increased arginase activity is involved in airway sensitivity in smokers [35]. Overall, elevated arginase activity may be one of the mechanisms underlying both pulmonary arterial dysfunction and bronchial dysfunction [36].

Studies with L-arg supplementation have shown inconsistent effects on both systemic and pulmonary endothelial functions [37-39]. In the present study, L-arg supplementation reduced the dysfunction in ED^+^ patients but did not restore a normal vasodilatory response; this finding suggests an absolute lack of substrate in these individuals. The lack of complete restoration of a normal vasodilatory response might involve (amongst others) additional mediators of pulmonary tobacco associated- endothelial dysfunction, such as increased activation of the endothelin (ET)-1/ET-A pathway (as we have previously reported [5]). In line with the recent literature, the ED^+^ and ED^−^ subgroups did not differ significantly in terms of expression of NOS3 (the arginases’ competitor for NO production) in the pulmonary vasculature [40]. The exact relationship between cigarette smoke and NOS3 expression/activity is still subject to debate. In animal studies, smoke exposure has variously led to a decrease or an increase in NOS3 expression [41-43]. Given that NOS3 is tightly regulated by post-translational lipid modifications, protein-protein interactions and protein phosphorylation [44], its protein expression levels are not correlated with its activity. The fact that genistein (a strong potentiator of NO production via NOS3 activation) did not enhance vasorelaxation in ED+ subjects suggests that impaired NOS3 activity does not have a role in endothelial dysfunction in that context.

## Conclusions

Arginase I overexpression and activity may be involved in tobacco-induced pulmonary endothelial dysfunction. The present results are of interest because they suggest that increased arginase activity may be one of the mechanisms underlying both pulmonary arterial dysfunction and bronchial dysfunction. Further research must explore the effects of combined L-arg supplementation and arginase inhibition and assess the potential value of these approaches in resolving endothelial dysfunction in the setting of tobacco exposure.

## Abbreviations

Ach: Acetylcholine
COPD: Chronic obstructive pulmonary disease
NO: Nitric oxide
NOS: Nitric oxide synthase
L-arg: L-arginine
NorNOHA: N-hydroxy-nor-L-arginine
ED^+^: Subgroup with endothelial dysfunction
ED^−^: Subgroup without endothelial dysfunction
FEV1: Forced expiratory flow in 1 second
GOLD: Global initiative for obstructive lung disease
